# Supplementary material for: Interindividual- and blood-correlated sweat phenylalanine multimodal analytical biochips for tracking exercise metabolism
Source: Nat Commun. 2024 Jan 20;15:624. doi: 10.1038/s41467-024-44751-z (PMC10799919; doi:10.1038/s41467-024-44751-z)
Supplement: Supplementary file 1 — Supplementary Information [file 41467_2024_44751_MOESM1_ESM.pdf]

Interindividual- and Blood-Correlated Sweat Phenylalanine Multimodal Analytical Biochips for Tracking Exercise Metabolism

Bowen Zhong[1,2], Xiaokun Qin[1,2], Hao Xu[1,2], Lingchen Liu[1,2], Linlin Li[1,2], Zhexin Li[1,2], Limin Cao[3], Zheng Lou[1,2], Joshua A. Jackman[4], Nam-Joon Cho[5] & Lili Wang[1,2]*

1. State Key Laboratory for Superlattices and Microstructures, Institute of Semiconductors, Chinese Academy of Sciences, Beijing 100083, China

2. Center of Materials Science and Optoelectronic Engineering, University of Chinese Academy of Sciences, Beijing 100049, China

3. Tianjin Key Laboratory of Lung Cancer Metastasis and Tumor Microenvironment, Tianjin Lung Cancer Institute, Tianjin Medical University General Hospital, Tianjin 300052, China

4. School of Chemical Engineering and Translational Nanobioscience Research Center, Sungkyunkwan University, Suwon 16419, Republic of Korea

5. School of Materials Science and Engineering, Nanyang Technological University, 637553, Singapore

Correspondence and requests for materials should be addressed to L. L. Wang (liliwang@semi.ac.cn).

Supplementary Note 1- Supplementary Note 3.

Supplementary Fig. 1- Supplementary Fig. 33.

Supplementary table 1- Supplementary table 3.

Supplementary Video 1- Supplementary Video 4.

Supplementary Note 1. Characterization of the PANI-based MIP electrode

Surface morphological analysis. As shown in Supplementary Fig.5, The Field-emission Scanning Electron Microscope (FSEM) studies of MIP electrodes have observed that the surface of polymer becomes rough and porous after the extraction of Phe and the electro-degradation. This surface morphological change was also verified by Atomic Force Microscopy (AFM). As illustrated in Supplementary Fig.6, the surface roughness of both E-MIP and E-NIP electrodes increased due to the degradation of PANI chains, while the E-MIP electrode increased more significantly due to the extraction of the imprinted Phe.

FTIR-ATR spectroscopy. The Fourier transform infrared attenuated total reflectance (FTIR-ATR) spectroscopy was employed to examine the change of surface functionalities before and after electroelution. After measuring the gold electrode as background (100 scans), the covered films including MIP, NIP, E-MIP, and E-NIP were characterized using 100 scans at 8 cm^{-1} resolutions (Supplementary Fig. 7). All characteristic frequencies of each electrode are summarized in Supplementary Table 2. Compared with NIP and E-MIP, the FTIR-ATR spectrum obtained for MIP showed an additional band at 1693 cm^{-1} corresponding to C=O stretching of carboxyl, which confirms the successful imprinting of the Phe molecule on the MIP electrode as well as the complete extraction of the imprinted Phe by the electro-elution procedure. The ratio of the intensity of band at 1511 cm^{-1} to that of the band at 1596 cm^{-1} indicated the

portion of over-oxidized quinoid rings elevates in the PANI matrix after the electro-elution for both MIP and NIP[1,2]. The band at 1103 cm^{-1} also increased due to the increased quinonimine content in the over-oxidized electrodes (E-MIP and E-NIP)[3]. Furthermore, compared with MIP and NIP, the electrodes after electro-elution showed an additional band at 1658 cm^{-1} assigned to C=O stretching of benzoquinone, revealing that the electro-elution produces benzoquinone endgroups on the E-MIP and E-NIP electrodes. These functional groups are vital for the electro-oxidation of Phe, which has also been demonstrated by the subsequent theoretical molecular simulation.

Preparation and optimization of MIP electrodes. As shown in Supplementary Fig. 11a,b, the CV scans after electro-polymerization and the MPS scans during electro-elution of two electrodes (MIP and NIP) were distinct. Due to the introduction of Phe as the template molecular, the first CV scans for the prepared MIP electrode had an additional oxidation peak around 0.5V compared to NIP. Moreover, as the electro-elution progresses, the MPS current on MIP electrodes was greater than that of NIP, revealing an additional increase in surface roughness of MIP electrodes resulted from the detachment of Phe molecule form the MIP matrix. Therefore, the switch number of MPS scans for electro-elution is a key optimization parameter affecting the sensing performance of the MIP electrode. Here, the peak current of E-MIP-based Phe sensor increased rapidly with the increase of switch number initially, then gradually stabilized after 40 switches, and eventually decreases rapidly

(Supplementary Fig. 11c).

Preparation of the PPY-based MIP electrode for comparison. The electro-polymerization synthesis method for PPY-based MIP electrode referred to the reported study.[4] In brief, the polymerization solution was prepared by dissolving 5 mM Phe as template and 50 mM pyrrole into 1×PBS (pH 7.0). Before electro-polymerization, the thermally evaporated gold electrode at the WE zone was drip-coated and cleaned with piranha solution (H_2SO_4 : H_2O_2 = 7:3 v/v). Then, the electro-polymerization was conducted by means of CV from -1.2 to 1 V versus SCE for 30 cycles at a scan rate of 500 mV/s. The imprinted molecules were extracted by soaking the electrode into an acetic acid/methanol mixture (7:3 v/v) for 1h. Subsequently, the resulting electrode was immersed into 1×PBS (pH 7.0) for repetitive CV scans (0.4–1 V at a scan rate of 50 mV s^{-1}) until a stable response was obtained.

EIS analysis. Electrochemical impedance spectroscopy (EIS) measurements were performed at their corresponding OCP using a sinusoidal potential perturbation with an amplitude of 0.05 V and frequency range from 0.01 to 100,000 Hz in 1×PBS (pH 7.0) containing 200 μM Phe (Fig. 2f) or 0.2 M KCl solution containing 5 mM $[Fe(CN)_6]^{3-/4-}$ redox probe (Supplementary Fig. 12). Impedance data were represented as Nyquist plots.

The semicircle diameter of Nyquist plot gives a value of charge transfer resistance (R_{CT}) that reveals electron transfer kinetics at the electrode interface.[5] As shown in Supplementary Fig. 12, R_{CT} increased as the gold

electrode was covered with the electro-polymerized films (MIP and NIP). After electro-elution, R_{CT} decreased due to the degradation of the PANI chain, while the E-MIP decreased more significantly than the E-NIP due to the extraction of the imprinted Phe. Moreover, the diffusion process referred to as Warburg behavior exhibits a straight line, whose slope can be used to qualitatively explain Warburg impendence (Z_w) associated with the diffusion of the electroactive species from the solution towards the electrode surface[6,7]. As shown in Fig. 2h, for the MIP electrode, the fitting slope was a large negative value indicating that it was coated with an insulating layer, effectively blocking electron transfer at the electrode/solution interface. In contrast, for the E-MIP electrode, the fitting slope is -0.88 indicating that it has a smaller Z_w closed to -1 (an ideal phase angle of 45°).

Effect of pH on the DPV peak potential change of Phe sensors. The values of peak potential were shifted to more negative potential with the increase of pH from 7.26 to 10.91 (Supplementary Fig. 16), revealing that proton involves in the electro-oxidation reaction[8]. By fitting the plot of peak potential versus pH, the obtained slope of 48 mV pH^{-1} is close to the value 59 mV pH^{-1}, indicating that an equal number of electrons and protons are involved and transferred in this electro-oxidation of Phe. The reaction mechanism may be as follows:

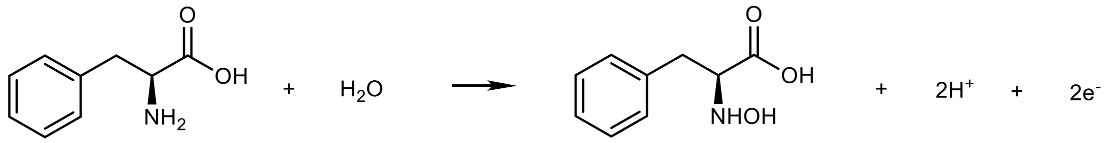

Supplementary Note 2. Theoretical molecular simulations of the PANI-based MIP electrode

PANI degradation model and resulting structures for simulation. PANI films formed on gold electrode can be anodically electro-degraded with time. The bulk of the PANI dissolves uniformly resulting in greater porosity and thinner thickness, while creating quinone endgroups in the matrix and producing soluble benzoquinone[9-11]. Here, to achieve the electro-elution of templates in PANI based MIP and simultaneously prevent the failure of the imprinting effect caused by rapid electro-degradation, we conducted the electro-degradation of PANI based MIP in a neutral solution environment (PBS) with a low degradation rate. According to the above FTIR-ATR analysis, the degradation in the work produced quinoid rings and benzoquinone endgroups[3]. Thus, the PANI degradation model here is similar to that in an acidic environment, as illustrated below[12].

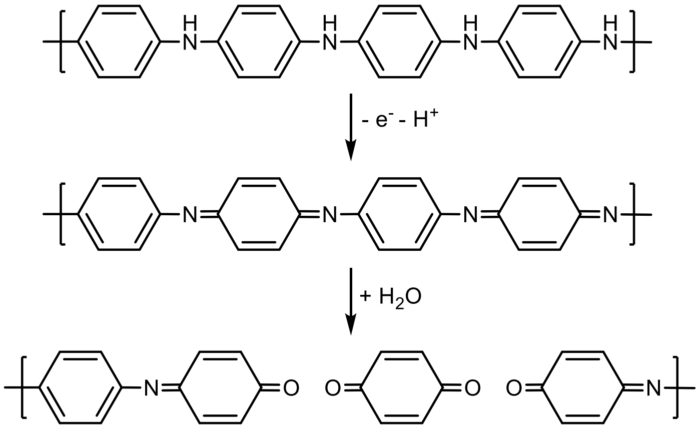

Taking these three structures as the polymer chain of PANI-based MIP for theoretical molecular simulations, they were named PANI_re, PANI_ox, and PANI_ov respectively from top to bottom.

Molecular interaction simulation between PANI and Phe. All geometric optimizations have been carried out by density functional theory (DFT) using the B3LYP hybrid functional with Grimme's dispersion correction of D3 version (Becke-Johnson damping). The standard 6-31G(d,p) basis set for C, H, N, and O atoms was used. PANI with eight repeating units was chosen as ligands to mimic before the degradation of PANI for forming the structure of PANI_re. During degradation, eight units with the alternating repetition of four benzenoid rings and four quinoid rings was chosen to mimic the structure of PANI_ox. After degradation, a combination of four and three units with alternating benzenoid and quinoid rings was chosen to mimic the structure of PANI_ov, because the original chain of PANI breaks. Here, the long polymer chains were used for accurate calculations of binding energies, efficient simulation of the molecular imprinting process, and further electrochemical behavior simulation. Using the Monte Carlo method in combination with the PM3 level of the semi-empirical theory, the most stable conformers of the above three structures of PANI as the host and zwitterionic Phe as the target analyte were estimated[13]. After that, Bonding energies between ligands and targets were calculated with the typical formula 1:

$$\Delta E = E_{\text{Ligand\_target}} - (E_{\text{Ligand}} + E_{\text{target}}) \quad (1)$$

The DFT simulated bonding energies and conformers of three ligand-target complexes were demonstrated in Supplementary Fig. 8. The energies of these molecular interaction between the ligand_target were estimated to be -19.40, -

14.66, and -12.30 kcal mol^{-1} for PANI_re, PANI_ox, and PANI_ov with Phe respectively. Apparently, after electro-elution, the binding of Phe to the PANI chain becomes weaker revealing the extraction of target molecules. This simulation suggests the mechanism of the electro-elution of Phe from the electrode to form the E-MIP upon controlled degradation, which maintains weak Phe interaction for further Phe sensing[14].

Intermolecular simulation for electrochemical behavior of Phe on electrodes. The electron transfer (Δq, the electrode obtains electrons as a positive sign) between Phe and electrodes was calculated under an external electric field for simulating the electrochemical behavior using the extended charge decomposition analysis (ECDA). As shown in Supplementary Fig. 9, the previously calculated conformers (PANI_ox_Phe and PANI_ov_Phe) were used here to mimic the MIP and E-MIP electrodes respectively. A single three units with alternating benzenoid and quinoid rings (nPANI_ov_Phe) was chosen to mimic the PANI-E-NIP electrode due to its fewer binding sites compared to the PANI-E-MIP electrode. In addition, the conformer (PPY_re_Phe) used for mimicking PPY-E-MIP electrode was constructed based on the relevant reference[12]. For gold electrode, A metal cluster model containing 31 gold atoms (Au_{31}) arranged in two layers of 19 and 12 atoms was used to model the Au(111) surface[15]. The LANL2DZ basis set and its corresponding effective core potential was used for describing the gold metal atoms[15]. Approximate solvent effects of water were taken into consideration in geometry optimization. In the absence

of an external electric field, all electrodes had low Δq values interacted with Phe show that the adsorption of Phe on all electrodes is a physisorption process in nature (Supplementary Fig. 9a).

To investigate the effect of external electric field on electrons transfer, an external electric field of 0.01 a.u. is applied in the normal direction to the electrode. This situation corresponds with experimental electrode potentials positive to the potential of zero charge. Notably, the adsorption behavior of Phe at the electrode surfaces was simulated with or without the external electric field, where these two configurations of Phe at the gold electrode are analogous to that reported in the literature (Supplementary Fig. 9b,c)[16], indicating that the electrode models we proposed has certain rationality. Importantly, the E-MIP electrode exhibits the greatest Δq of 3.924e, followed by the E-NIP electrode with Δq of 3.013e, the PPY electrode with Δq of 2.861e, the gold electrode with Δq of 2.757e, and the MIP electrode with smallest Δq of 0.103e (Supplementary Fig. 9c-g). This calculation results showed the E-MIP electrode has a higher electro-catalytic activity for oxidizing Phe.

Potential mechanism of electro-catalytic oxidation. All of the aforementioned proofs pointed out that the benzoquinone endgroups on the E-MIP electrode surface are vital for the electro-oxidation of Phe. The specific mechanism is most likely due to the fact that the benzoquinone endgroups are the reactive sites for electrophilic (positive potential region, electron-poor) attacks, and the amino group as well as the -OH fraction of carboxyl group are

the reactive sites for nucleophilic (negative potential region, electron-rich) attacks[17]. This conjecture was confirmed by the molecular electrostatic potential (MEP) analysis finished by Multiwfn (Fig. 2e, inset). Two diametrically opposed properties reinforced each other under the effect of external electric field, resulting in a high Δq value.

Supplementary Note 3. Characterization of the multipurpose microfluidics

Estimation for injection rates of syringe pump. The injection rate of syringe pump was calculated and determined in advance to match the actual sweating of human body. Here, the inlet area of microfluidics was 75.36 mm^2 according to our CAD design. Generally, the sweat rate of human body is about 200 to 2000 g m^{-2} h^{-1} during exercise[18].After defining the density of sweat to 1 g mL^{-1}, the injection rate should be set in the range of 0.25 to 2.5 μL min^{-1}.

Fluid capacity of microfluidics for collecting sweat. For the chamber and inlets of microfluidics, the fluid capacity was about 24 μL without the embedded filter paper. After the introduction of filter paper, it is important to accurately calibrate the changed liquid capacity by considering the increase in the swelling thickness of the filter paper (Supplementary Fig. 17) and its intrinsic water absorption volume for subsequent colorimetric sweat loss sensing. When the filter paper was placed in the chamber, the fluid capacity was reduced to 16 μL by subtracting the occupied volume caused by water absorption and expansion (11 μL) as well as adding its water absorption volume (3 μL). This reduction in chamber volume capacity facilitates the rapid sweat collection for sweat

sensing. For the elongated serpentine outflow channel, the height and width of the channel cross-section were 170 μm and 240 μm respectively, and thus the path length of a meandering serpentine channel was designed to be 24.5 mm, resulting in a fluid capacity of just 1 μL. Therefore, the fluid capacity of 21 repeating serpentine outflow channels was 21 μL here.

Numerical simulation and comparison of filling process. The liquid filling behaviors over time in the chamber with or without an embedded filter paper were simulated to mimic sweat sampling using a computational fluid dynamics module (Laminar Flow, Level Set interface) in COMSOL Multiphysics 6.0 (Supplementary Fig. 18). Each inlet flow rate was set as 1/6 μL min^{-1} to match the sweat rate (2 μL min^{-1}) of human forehead measured by our microfluidics with 12 inlets, and the outlet condition adopted was one atmospheric pressure. The fluid flow properties were assumed to be the same as water since the composition of sweat is 99% water. Porous Media Domain was used to study the effect of filter paper on the filling process. As shown in Supplementary Fig. 16a,b and Video 3, the fluid could fill the chamber with an embedded filter paper in 8 min, which is 3 min faster than the case without filter paper. This result agrees with the above calculated volume difference of two different chamber with or without filter paper. The water/air interface was reflected by an isoline at a water phase volume fraction of 0.5 to study the movement of the interface under water filling. Moreover, the embedded filter paper will not affect the flow rate field in the chamber, but the flow lines will be more uniform and smooth

(Supplementary Fig. 18c,d).

Numerical simulation and comparison of refreshing time. The old solute concentration was continuously refreshed by the new inflowed solute concentration in the chamber during sweat sampling. Such mass transport process analyses were performed using numerical simulation (COMSOL Multiphysics) by coupling Transport of Diluted Species interface and Laminar Flow interfaces. For the case without filter paper, this process was simulated by numerically solving Stokes equation for an incompressible flow coupled with convection-diffusion equation[19]. For the case with filter paper, the simulation of this process would be more complicated due to the mass transport in porous media, and needed to coupling mass balance equation for saturated porous media.

Here, the mass flow rate at each inlet was set to 1/6 mg min^{-1} with the non-slip boundary condition on all channel walls. The initial Phe concentration C_0 and supplied new Phe concentration C_1 were set as 100 μM and 50 μM respectively to simulate the concentration reduction measured in the subject's experiment. The concentration redistribution in the chamber is displayed at different time instances with or without filter paper (Supplementary Fig. 19), and the time for the average volume concentration of the chamber is also calculated according to the simulated concentration results of each point (Fig. 3d). For the case with filter paper, the time to reach 95% refreshing ratio of the C_1 is around 3 min, while that time is 8 min for the case without filter paper. This result shown

that the embedded filter paper can accelerate the refreshing process of concentration. Moreover, as shown in Supplementary Fig. 19 and Video 4, the concentration redistribution during mass transport was uniform and free of anomalous hard-to-transport areas in the filter-paper-embedded chamber compared to the case without filter paper.

Significance summary of the embedded filter paper. According to the argument from the above Supplementary Note and the main text, the embedding of filter paper in the chamber has the following functions. First and foremost, it buffers weakly acidic sweat to pH neutral and maintains the detection environment at a high ionic strength level in sufficient time for stable and reliable sweat Phe measurement. Second, it reduces the volume of sweat needed to fill the chamber for rapid sweat sampling and further sensing. Third, it enables to accelerate the concentration refreshing time and optimized the concentration redistribution behavior. All these functions contribute to the accurate and reliable Phe measurement by our sensors.

Design and principle of serpentine channel for flow visualization. The vertical microfluidic structure interconnected by the chamber was designed to implement the meandering serpentine channel in a controlled footprint. The principle of flow visualization is based on the remarkable difference in optical properties (reflectivity or transmittance) under visible light before and after sweat filling the channel. Specifically, after the top layer of PET was roughened by laser engraving, the prepared μ-dots microstructure on its surface was

suspended on the channel leading to a large reflectivity (small transmittance) stemmed from diffuse reflection (Supplementary Fig. 22). The sweat filling process largely eliminated total internal reflections from these μ-dots, thus remarkably modulating the optical properties (Supplementary Fig. 18a and Supplementary Video 2). Here, we choose PI as the interlayer of microfluidics, which not only isolates the inflow and outflow channels, but also serves as a dark background layer producing a more apparent color change for direct reading by naked eyes than the transparent PET (Supplementary Fig. 21b).

Computer vision algorithm for automatic sweat loss reading and comparative analysis

The photos used in the algorithm to automatically calculate sweat loss came from original snapshots of the system patch on the foreheads of eight subjects over a period of 10 to 20 min during exercise. All photos were converted to RAW format for eliminating artifacts. The algorithm based on traditional computer vision techniques identified the locations of relevant features, including the geometric outline and orientation of the microfluidic patch as well as the location of color-swatch variations of the visualized outflow channel within the outline. Sweat volumes could be determined by locating the interface between the white microchannels that have not changed to the background color and the background-color microchannels due to sweat filling. Sweat rates could be computed from sweat volumes and the time interval of picture capturing.

To verified the accuracy of colorimetric sweat loss measurement with distinct

visual color change, the sweat volume and rate measured by the automatic algorithm are compared to that visually read by users' naked eyes (Supplementary Fig. 24). The visually measured results are highly positively correlated with the algorithm results (Supplementary Fig. 24a, b). Calculated by a two-tailed t-test (Supplementary Fig. 24c), there is no significant difference between the two results ($P = 0.370$). Therefore, without the assistance of the automatic reading function, the sweat loss sensor with 0.5 μL volume resolution developed for this work can also measure accurate readings with naked eyes.

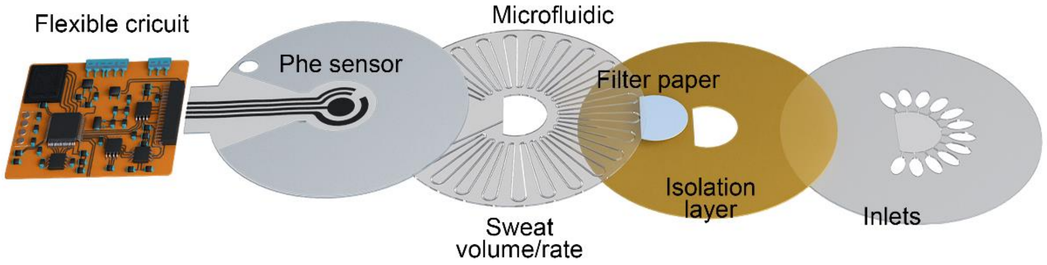

Supplementary Fig. 1 Illustration of wearable multimodal biochip for sensing multiple indicators.

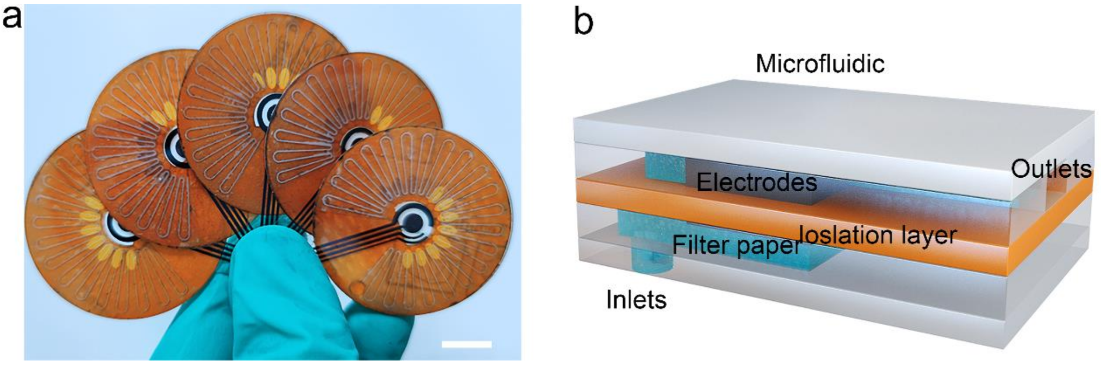

Supplementary Fig. 2 Illustrations of the integrated sweat sensor. a, Photograph of the Mass-produced scalable sweat sensors**.** Scale bar, 1 cm. **b**, 3D structure diagram of the sensor.

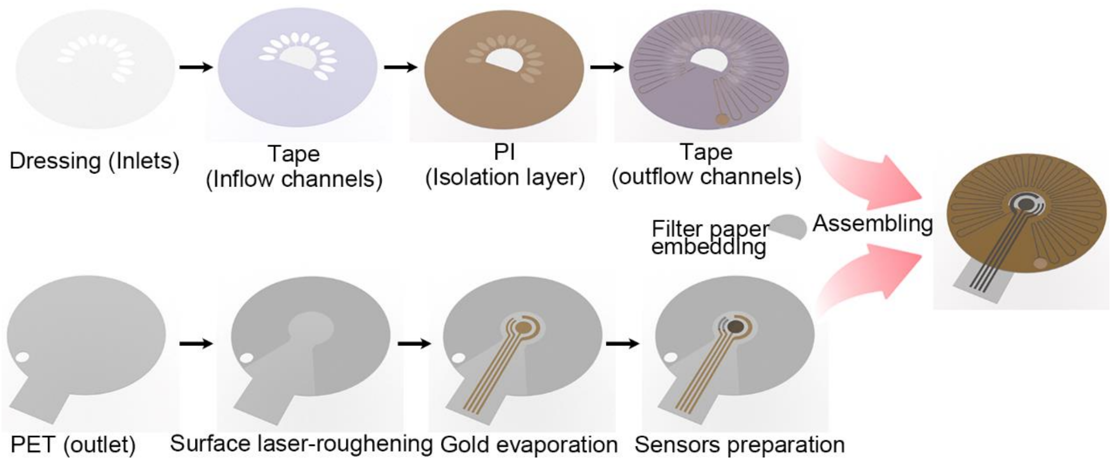

Supplementary Fig. 3 Fabrication procedures of wearable sweat Phe sensor.

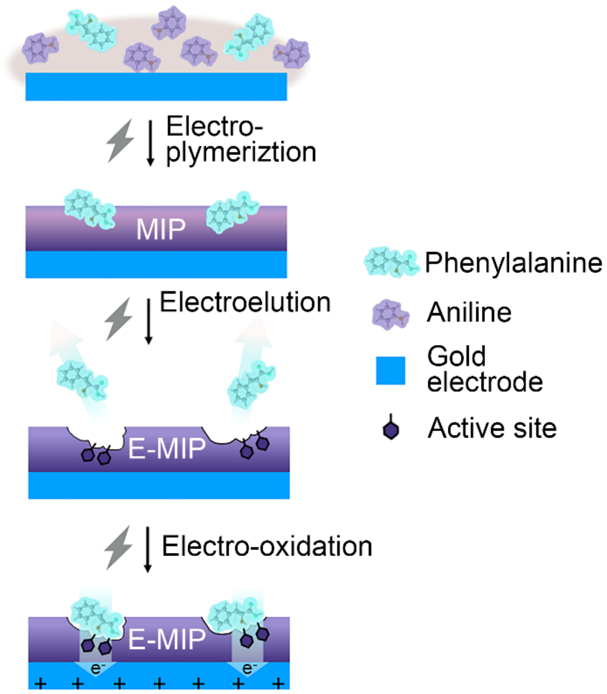

Supplementary Fig. 4 Schematic of the preparation procedure and sensing mechanism of the E-MIP-based Phe sensor.

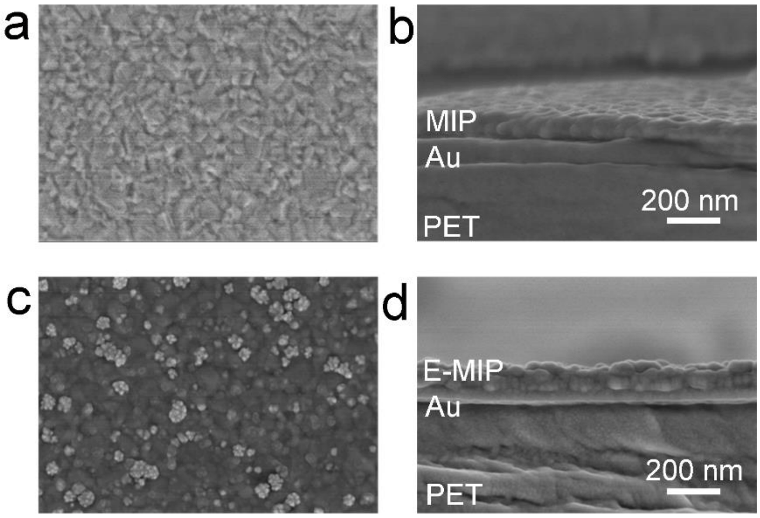

Supplementary Fig. 5 SEM images of the MIP and E-MIP electrode. a,**b**, Surface (**a**) and section (**b**) SEM images of a MIP electrode. **c**,**d**, Surface (**c**) and section (**d**) SEM images of an E-MIP electrode.

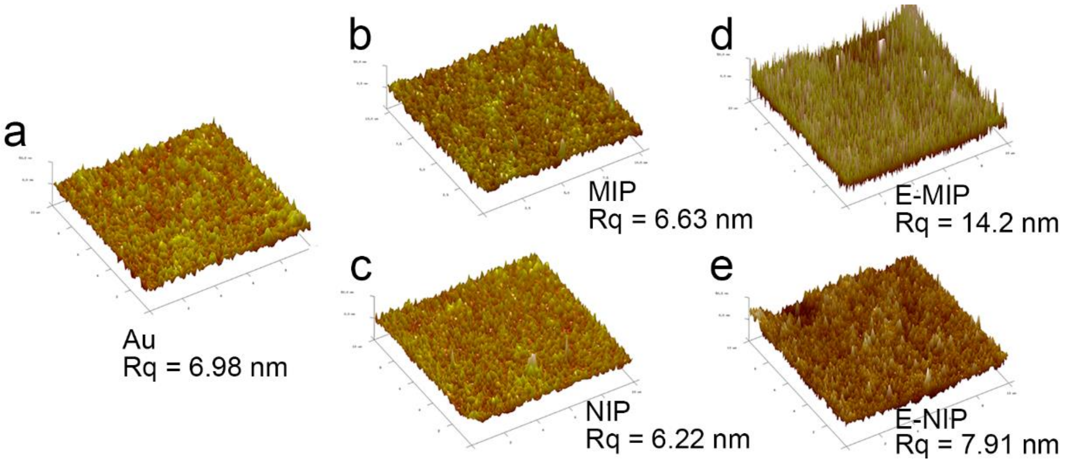

Supplementary Fig. 6 AFM images recorded using Height Mode for the PANI-based electrodes. **a**, gold electrode. **b**, MIP electrode. **c**, NIP electrode. **d**, E-MIP electrode. **e**, E-NIP electrode.

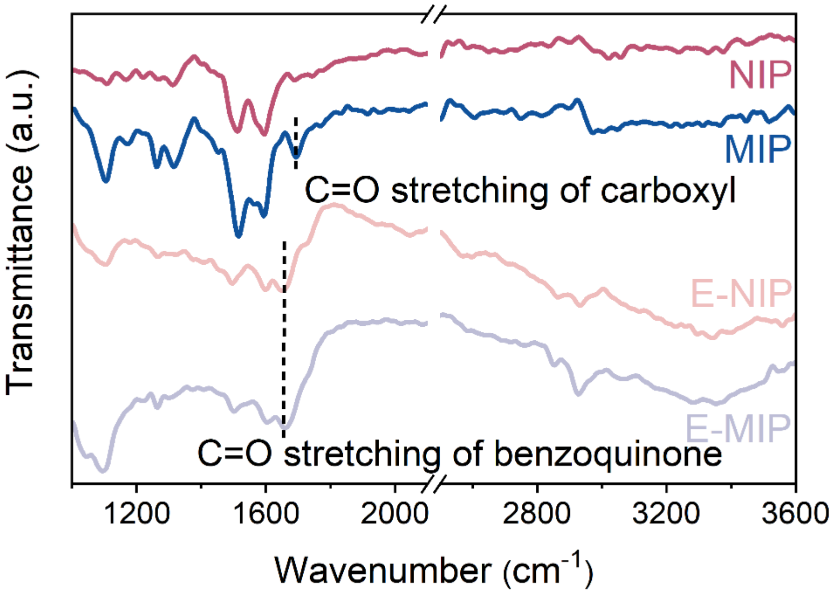

Supplementary Fig. 7 FTIR-ATR spectrum of the PANI-based electrodes.

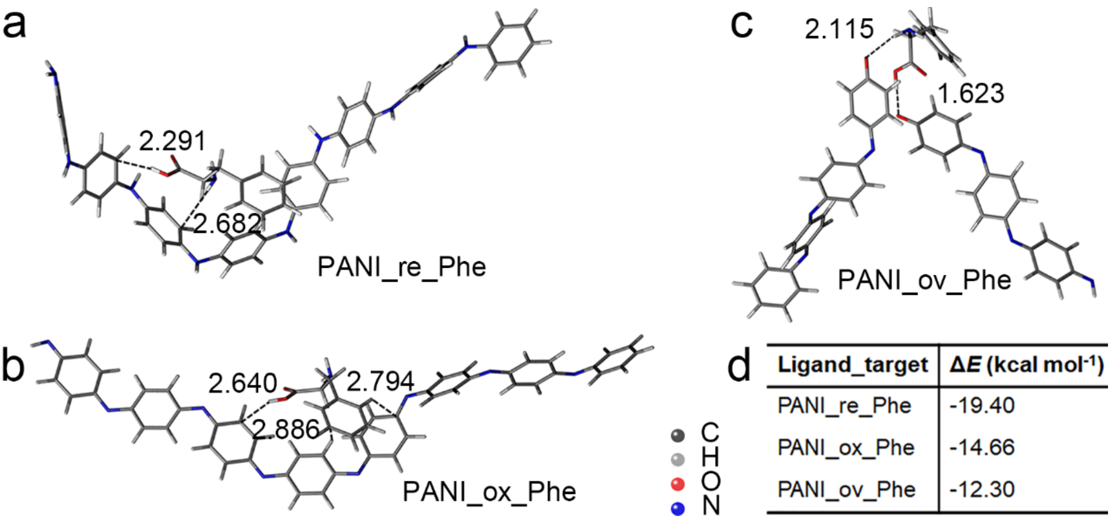

Ligand_target	Δ*E* (kcal mol^{-1})
PANI_re_Phe	-19.40
PANI_ox_Phe	-14.66
PANI_ov_Phe	-12.30

Supplementary Fig. 8 Molecular interaction simulation between a PANI chain and a Phe molecule. a-b, DFT-optimized geometries of the target (Phe) bonding with PANI_re (**a**), PANI_ox (**b**), and PANI_ov (**c**). **d**, DFT simulated bonding energies of the ligand_tagert complexes.

a

Electrode	Δq w/o an electric field	Δq w/ an electric field
Gold (Au_{31})	0.381e	2.757e
PPY (PPY_ox)	0.126e	2.861e
E-NIP (nPANI_ov)	0.034e	3.013e
MIP (PANI_re)	0.029e	0.103e
E-MIP (PANI_ov)	-0.099e	3.924e

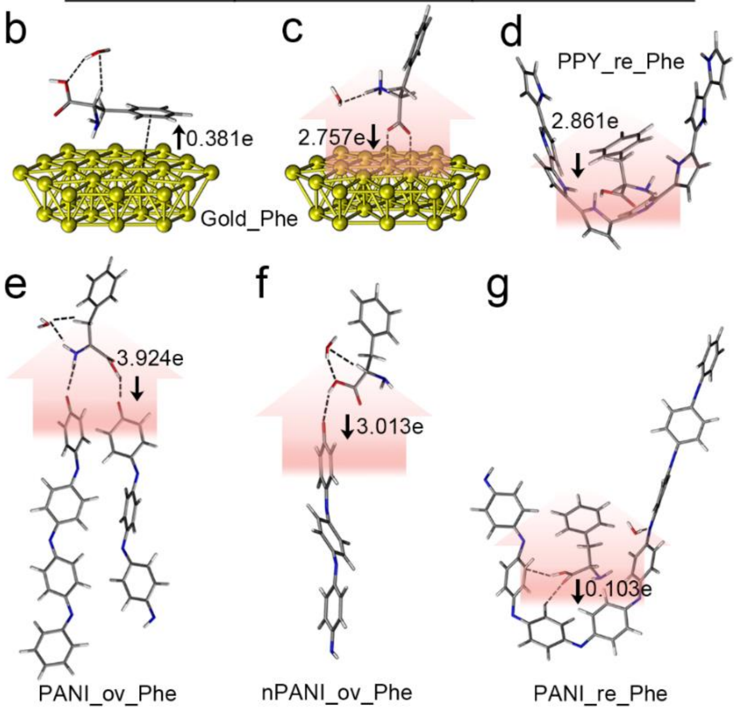

Supplementary Fig. 9 Intermolecular simulation and electron transfer calculation. a, Values of electron transfer (Δq) between five different electrodes and Phe w/ or w/o an external electric field. **b**, absorption behavior of Phe on the gold electrode. **c-g**, Electrochemical behavior and electron transfer of Phe on the gold (**c**), PPY (**d**), E-MIP (**e**), E-NIP (**f**), and MIP (**g**) electrodes. The red arrow represents the direction of the external electric field.

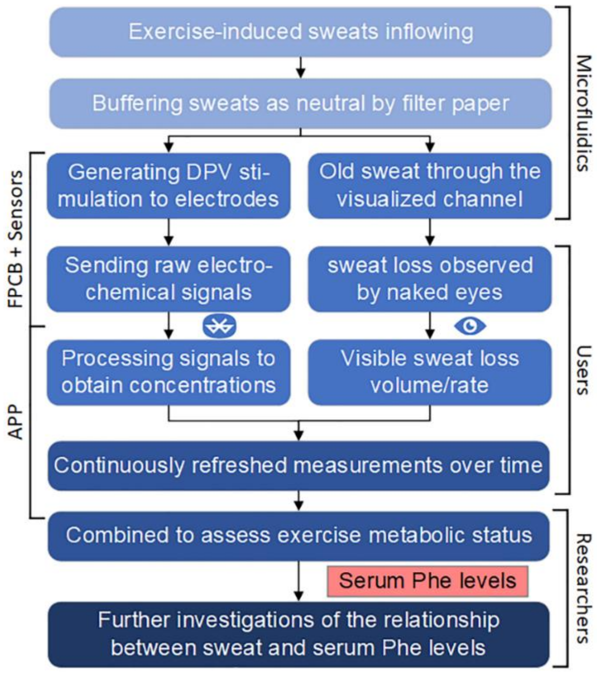

Supplementary Fig. 10 Workflow diagram of the overall wearable integrated system for healthcare applications.

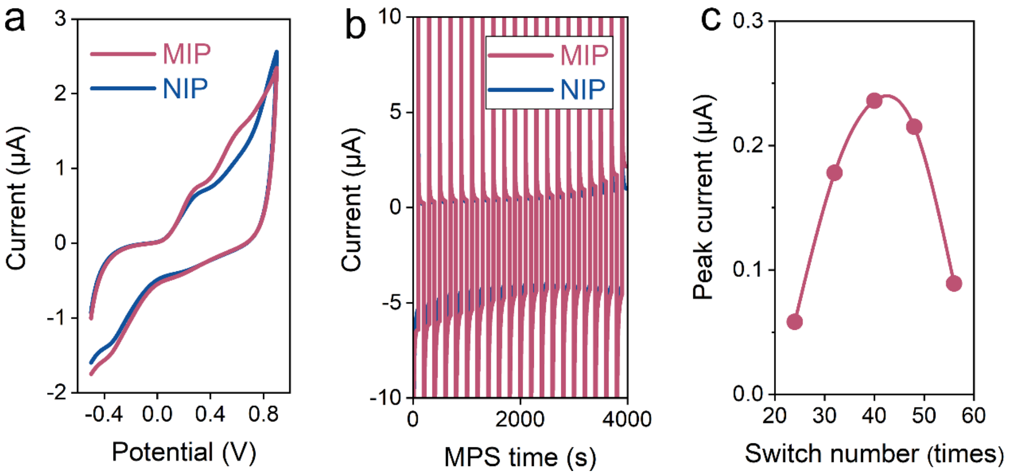

Supplementary Fig. 11 Preparation and optimization of MIP electrodes. **a**,**b**, Comparison of the MIP and NIP electrode at CV scans **(a)** after electro-polymerization and MPS scans **(b)** during electro-elution in PBS. **c**, Peak current of the Phe sensors in PBS containing 200 μM Phe varied from 24 to 56 MPS switch cycles.

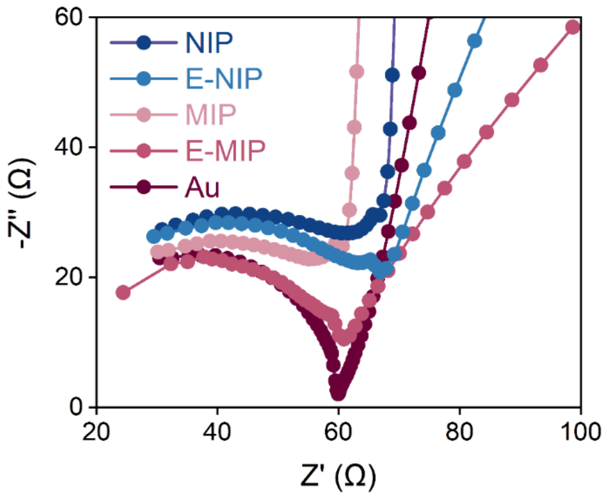

Supplementary Fig. 12 EIS Nyquist plots for five different electrodes in 0.2 M KCl solution containing 5 mM $[Fe(CN)_6]^{3-/4-}$ redox probe.

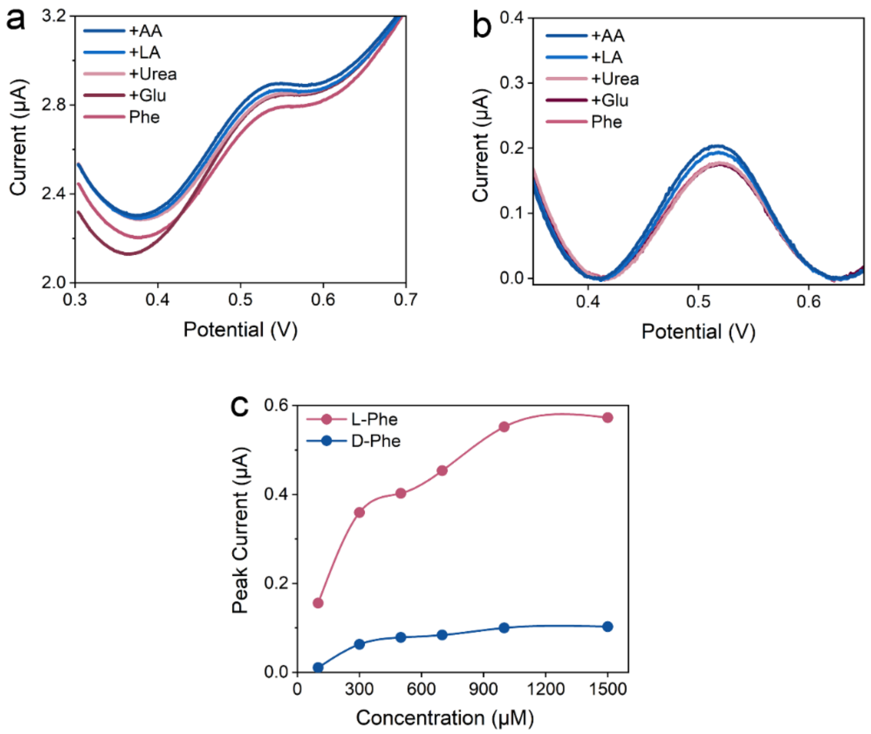

Supplementary Fig. 13 Evaluation of the Phe sensor selectivity. a,**b**, study of selectivity against common sweat interferents based on raw DPV data (**a**) and data after baseline correction (**b**). The following substances added in succession: 200 μM Phe, 100 μM glucose (Glu), 5 mM Urea, 5 mM lactate (LA), and 100 μM ascorbic acid (AA). **c**, Chiral recognition capacity for the L enantiomer (L-Phe) against the D enantiomer (D-Phe).

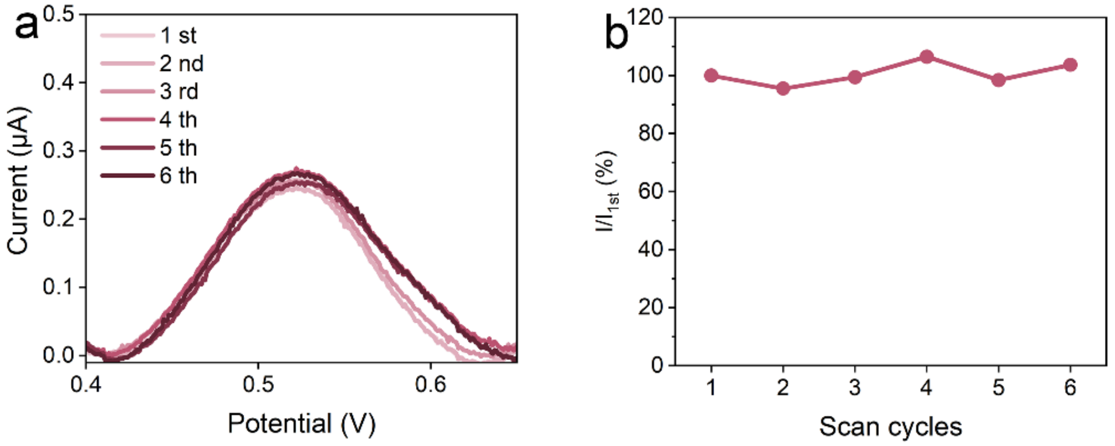

Supplementary Fig. 14 Reproducibility of the Phe sensor. a,**b**, DPV scans (**a**) and variation (**b**) of six continuous successive measurements using an E-MIP electrode in presence of 200 μM Phe.

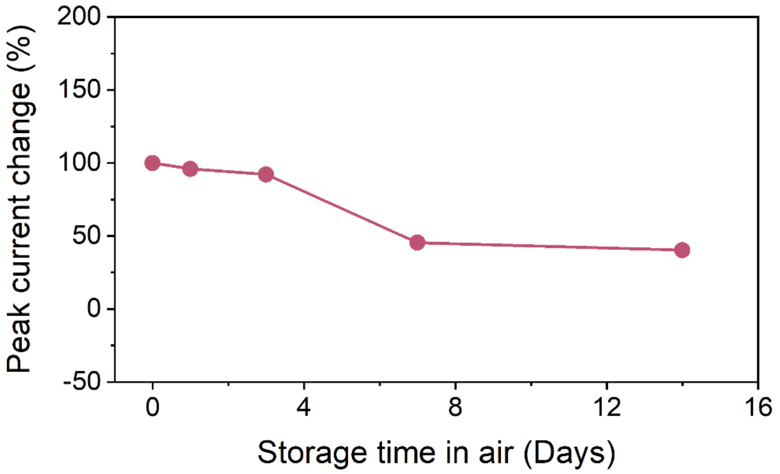

Supplementary Fig. 15 Evaluation of the Phe sensor storage stability. Long-term storage stability of the Phe sensor for detecting 200 μM Phe.

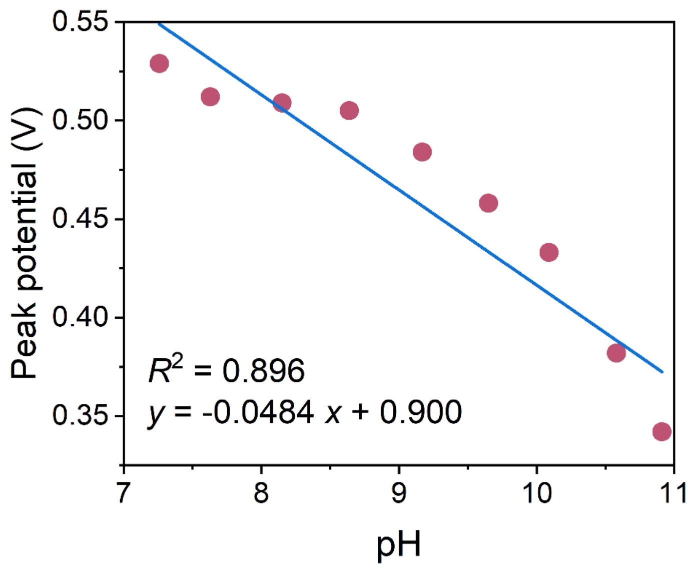

Supplementary Fig. 16 Effect of pH on the E-MIP electrode in DPV peak potentials. The solid line represents the linear fitting trendline.

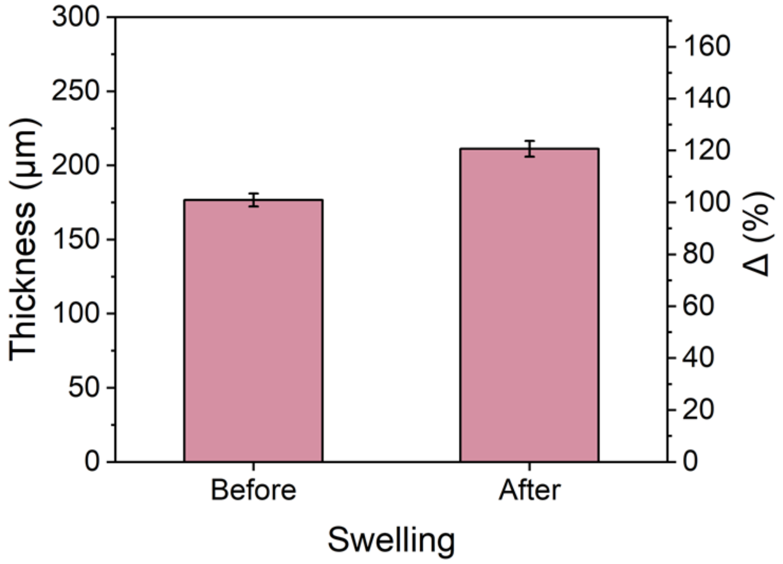

Supplementary Fig. 17 Thickness change of the filter paper before and after hygroscopic swelling (n = 4 independent experiments).

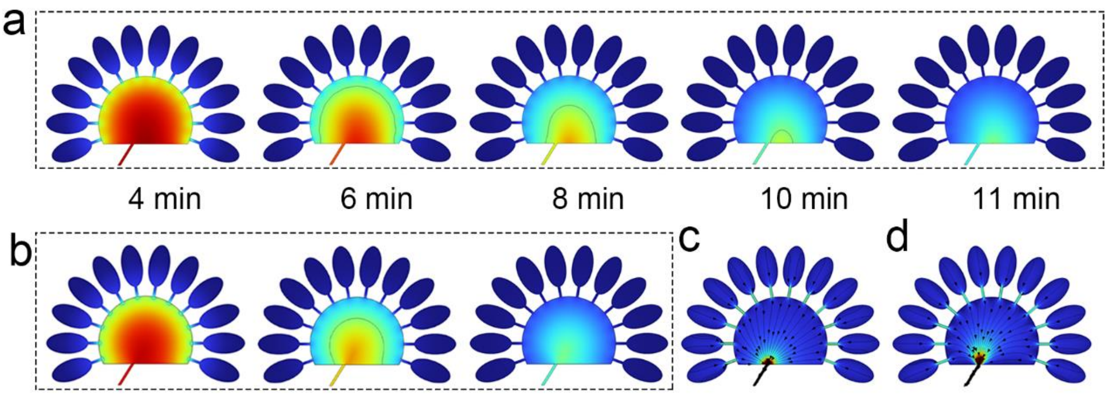

Supplementary Fig. 18 Effect of the embedded filter paper in the chamber on sweat filling process. **a**,**b**, Simulations of the fluid filling process in the chamber at different time instances without (**a**) or with (**b**) an embedded filter paper. **c**,**d**, Flow rate field and flow lines distribution in the chamber filled without (**c**) or with (**d**) an embedded filter paper.

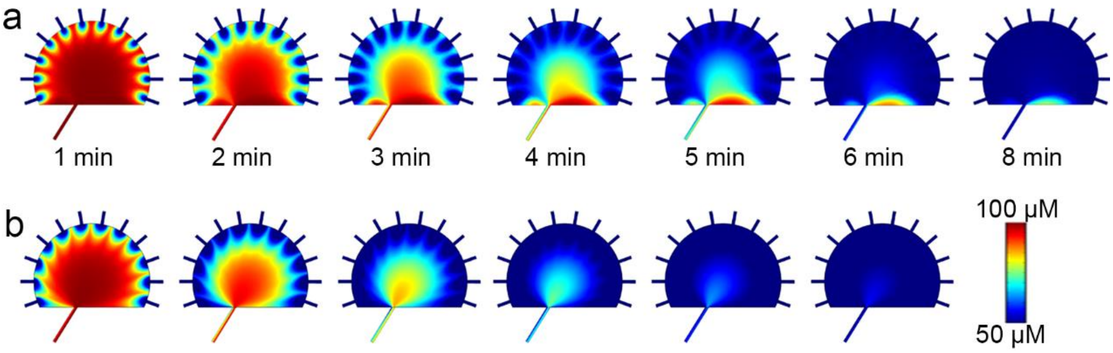

Supplementary Fig. 19 Effect of the embedded filter paper in the chamber on concentration refreshing process. Simulations of the redistributions of solute concentration in the chamber at different time instances without (**a**) or with (**b**) an embedded filter paper.

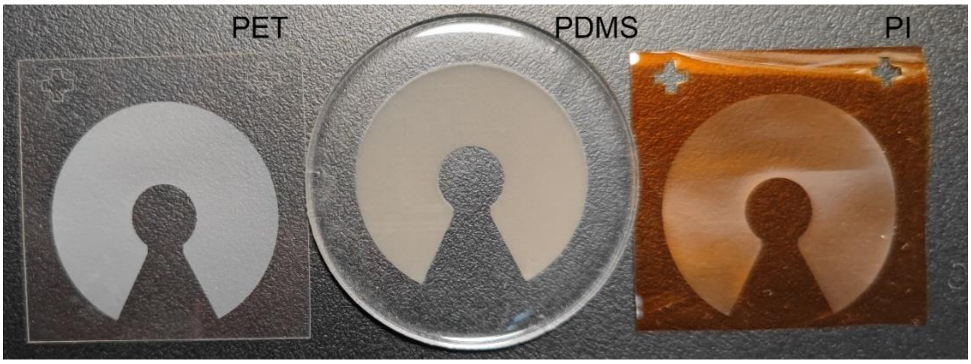

Supplementary Fig. 20 Photographs of different flexible substrates after surface roughening by laser engraving.

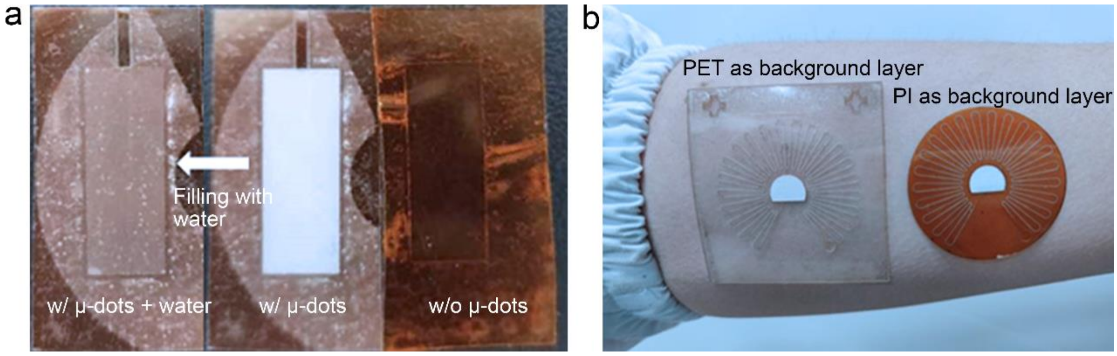

Supplementary Fig. 21 Design and principle of channel for flow visualization. a, Photograph of changes in optical properties before and after fluid filling. **b**, Photograph of comparison of PET and PI as the microfluidic background/isolation layer respectively.

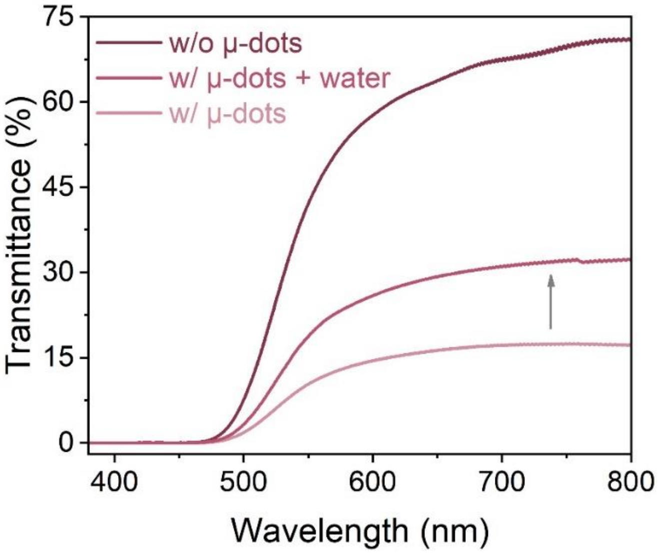

Supplementary Fig. 22 Optical transmittance of empty channels with or without μ-dots and filled channels with μ-dots.

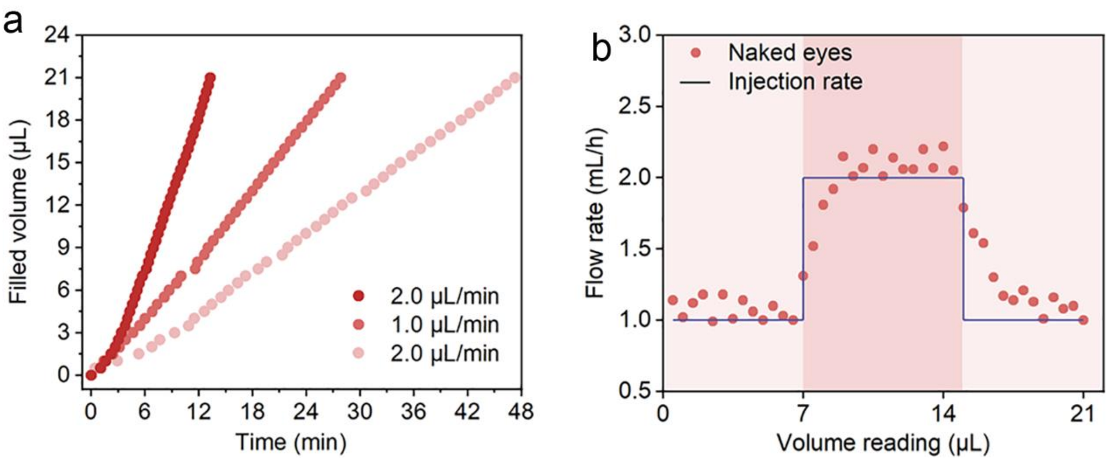

Supplementary Fig. 23 Characterizations of the outflow channel with flow visualization for flow rate measurement. a, Cumulative filled volume observed by naked eyes versus the time spent under varied pump flow rates of 2.0, 1.0, and 0.5 μL min^{-1}, respectively. b, Flow rate measurement during injection flow rate switching between 1.0 and 2.0 μL min^{-1}.

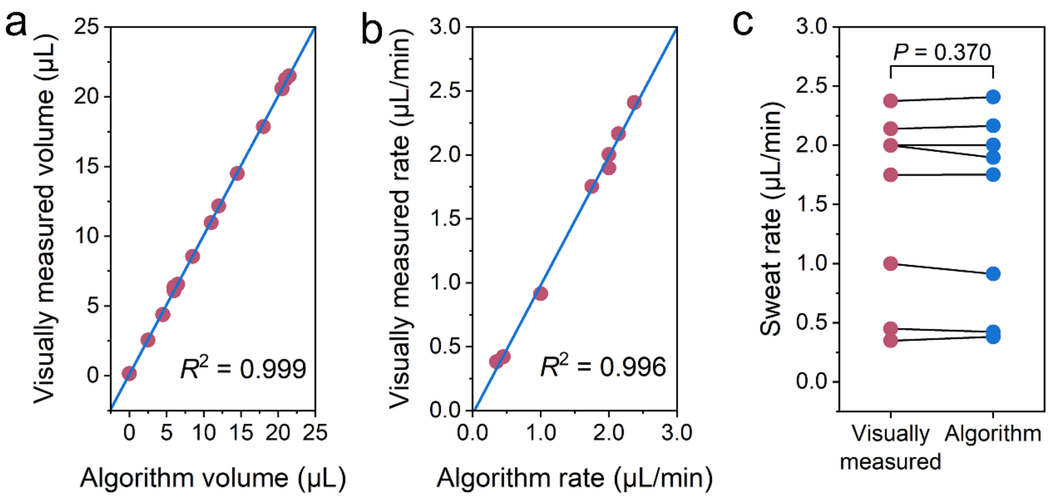

Supplementary Fig. 24 Comparison of the sweat loss values calculated from visual reading and algorithm. a,**b**, High-positively correlations between two methods for measuring sweat volumes (**a**) and sweat rates (**b**). **c**, Line sequence diagram of sweat rates measured by two methods. Statistical analysis based on a two-tailed t-test showed that there was no significant difference between these two groups of paired data (n = 8).

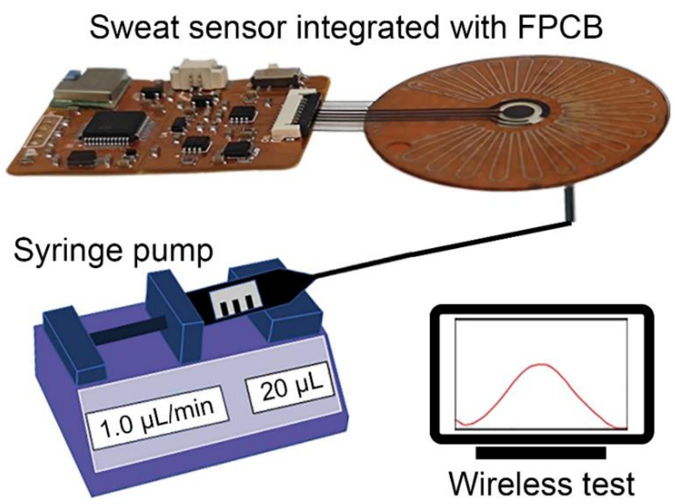

Supplementary Fig. 25 Experimental setup of the flow test to wireless sense the Phe levels in real raw sweat samples.

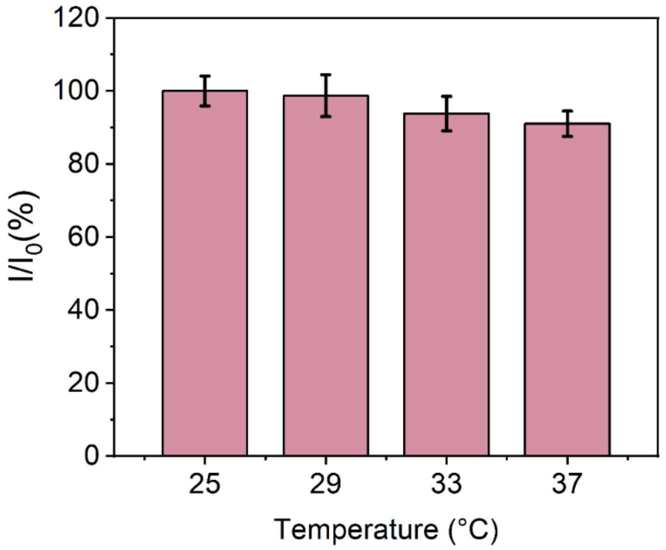

Supplementary Fig. 26 Performance of the Phe sensor at varied temperatures. (n = 3 measurements).

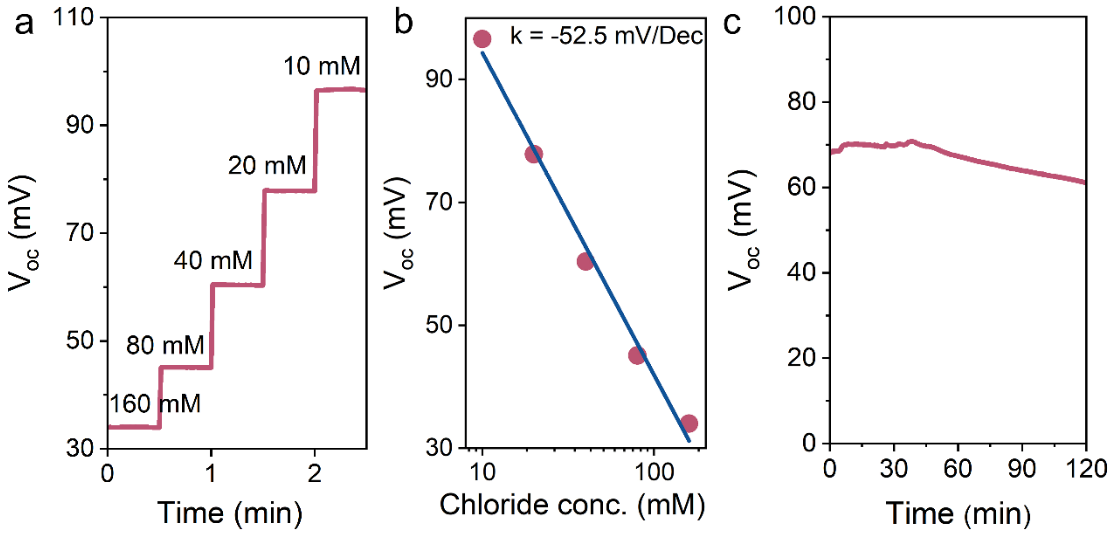

Supplementary Fig. 27 Characterizations of the chloride sensor. a,**b**, Open-circuit potential (V_{oc}) response curves (a) and corresponding Calibration curves (b) at varied chloride concentrations. The slope of the fitting line is -52.5 mV/Dec. **c**, Long-term stability in a real human sweat samples.

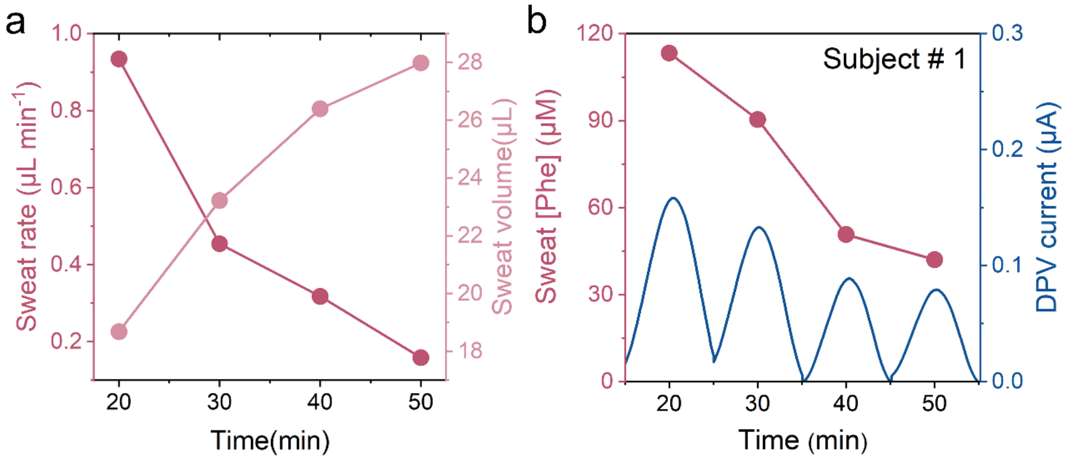

Supplementary Fig. 28 Real-time sweat monitoring during jogging. a, sweat loss (volume and rate) measured by the automatic reading algorithm. b, Phe concentrations along with corresponding DPV data from 0.4 to 0.6 V per scan obtained from the forehead of subject #1.

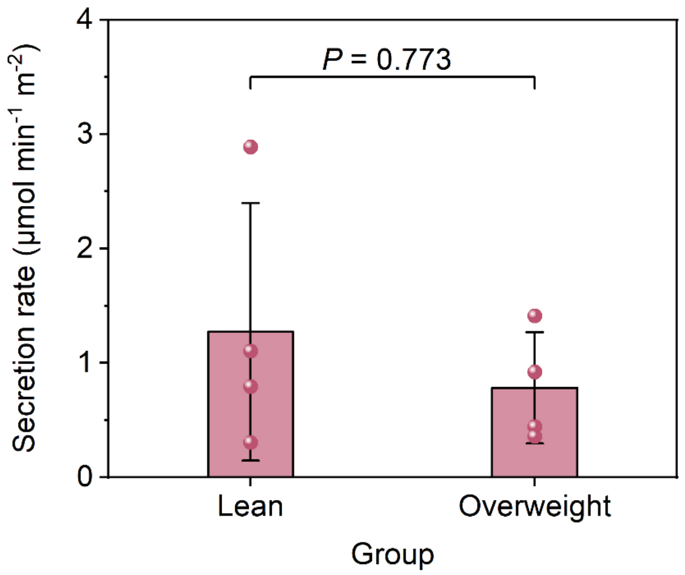

Supplementary Fig. 29 Comparison of sweat Phe secretion rate during exercise between lean and overweight group. (two-tailed Wilcoxon rank-sum test, W = 378; P = 0.773). The error bars correspond to the standard deviation (n = 4 samples). The center for the error bars represents the mean value.

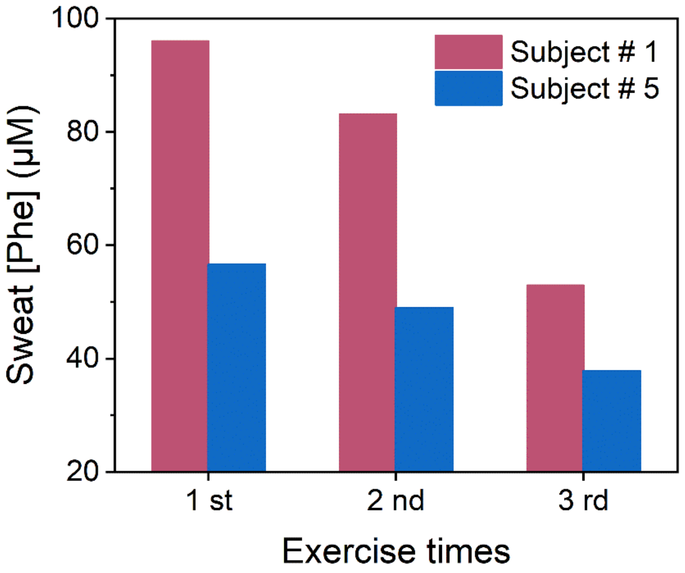

Supplementary Fig. 30 Changes in initial sweat Phe concentration of two subjects under multiple exercises. Sweat Phe concentrations were measured at the initial stage of exercise sweating (10 min).

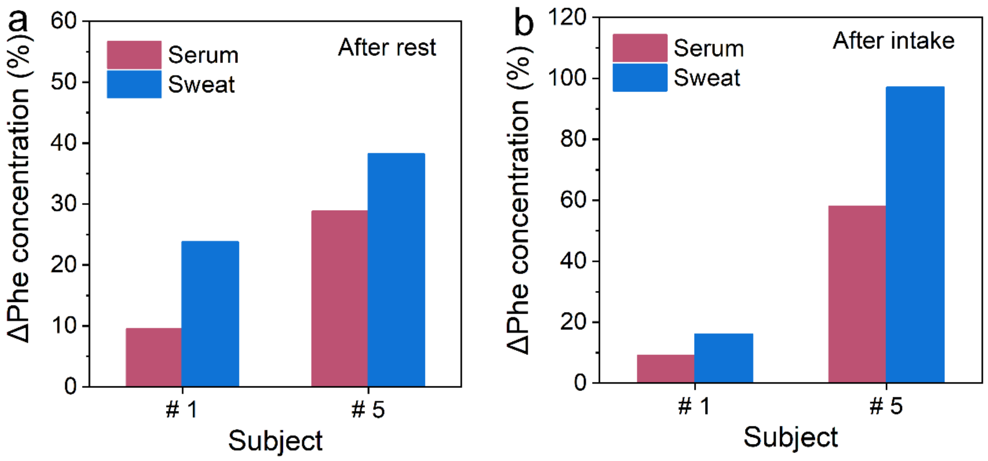

Supplementary Fig. 31 Comparison of percentage fluctuations in sweat and serum Phe levels between two subjects. a,**b**, Percentage fluctuation of the Phe (ΔPhe) level after intake (**a**) and rest (**b**) respectively. The raw data comes from Fig. 5c,d in the main text. ΔPhe takes the absolute value when it is negative.

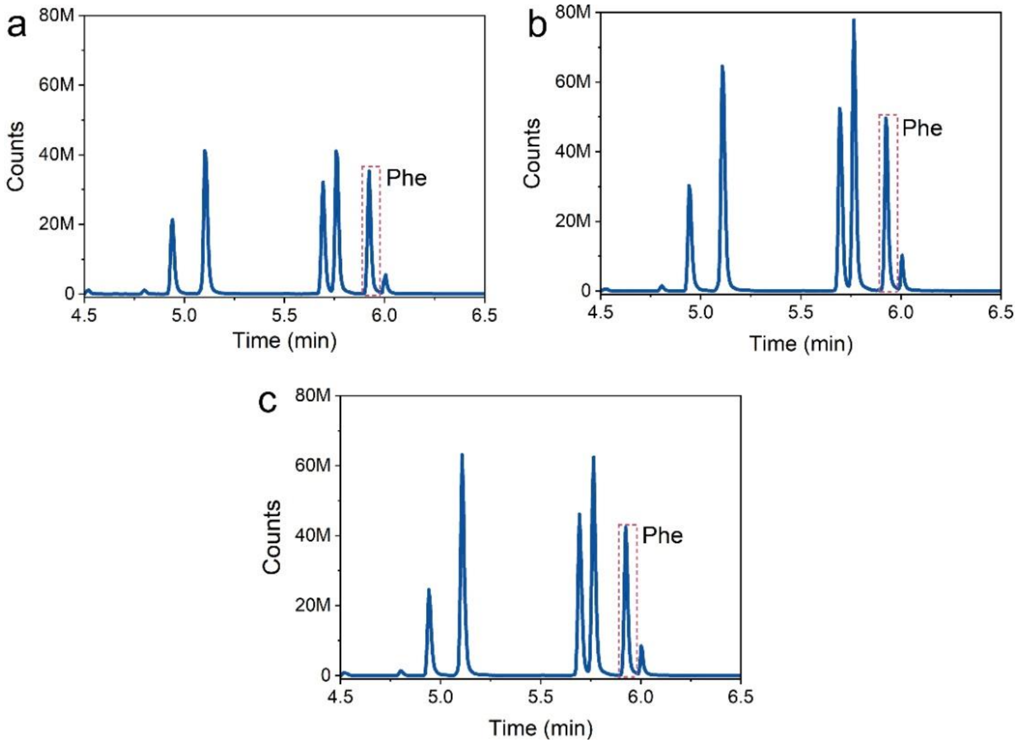

Supplementary Fig. 32 LC-MS analyses of the Phe levels in human serum samples. Spectra obtained from three serum samples taken from a subject after three consecutive exercises. **a**, sample collected before protein intake. **b**, sample collected one hour after protein intake. **c**, sample collected two hours after protein intake. Serum samples are diluted for 2 times. Characteristic peak of Phe is located at 5.92 min.

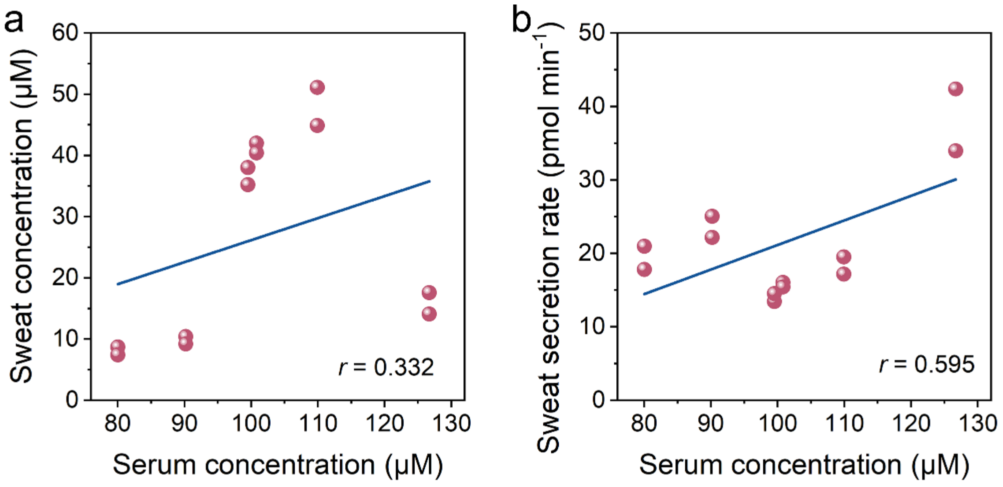

Supplementary Fig. 33 Interindividual correlations between sweat and serum Phe levels before (a) and after (b) sweat rate normalization in two subjects.

Supplementary Table 1. Comparison of recent portable Phe sensing devices.

Biofluid	Wearable form	Recognition element	Signal transduction technique	Direct detection	Human subject	Explore correlation with blood levels	LOD or lowest level studied (μM)	Ref.
Blood	No	Semisynthetic sensor proteins	luminescent	Yes	Yes, 40		1.01	[20]
Blood	No	Phenylalanine dehydrogenase	Colorimetric	Yes	Yes		66.6	[21]
Urine	No	Phenylalanine ammonia lyase	Colorimetric	Yes	Yes	No	60	[22]
Urine	No	Cell	Fluorescent	Yes	No	No	4.87	[23]
Saliva	Yes	Derivatization with 1,2-naphthoquinone-4-sulphonate	Electrochemical	No	Yes, 2	No	3.0	[24]
Sweat	Yes	PPY-based MIP and redox probe	Electrochemical	No	No	No	10	[4]
Sweat	Yes	OPD-based MIP and redox probe	Electrochemical	No	Yes, 2	No	5.0	[25]
Sweat	Yes	Electrocatalytically active PANI-based MIP	Electrochemical	Yes	Yes, 8	Yes	4.7	This work

PPY: polypyrrole; OPD: o-phenylenediamine; PANI: polyaniline

Supplementary Table 2. Characteristic frequencies of electrodes from FTIR-ATR

Wavenumber (cm^{-1})	Band characteristics	Electrodes
3351	N-H stretching	E-MIP; E-NIP
1693	C=O stretching of carboxyl	MIP
1658	C=O stretching of benzoquinone	E-MIP; E-NIP
1596	C=C stretching of benzenoid rings	MIP; NIP; E-MIP; E-NIP
1511	C=C stretching of quinoid rings	MIP; NIP; E-MIP; E-NIP
1261	Aromatic C−N stretching	MIP; NIP; E-MIP; E-NIP
1103	Imine C=N-H vibration	MIP; NIP; E-MIP; E-NIP

Supplementary Table 3. Body index and sweat information of eight male subjects

Group	Number	Body index			Exercise sweat information (10 to 20 min)		
		Height (m)	Weight (kg)	BMI (kg m^{-2})	Sweat rate (μL min^{-1})	Phe level (μM)	Secretion rate (μmol min^{-1} m^{-2})
Lean	1	1.83	71	21.2	0.382 (0.35)	56.7	0.30336
	2	1.80	70	21.6	1.752 (1.75)	125.8	2.88755
	3	1.67	58	20.8	0.422 (0.45)	143.5	0.79353
	4	1.75	60	19.6	0.914(1)	92.2	1.10374
Overweight	5	1.69	80	28.0	2.408 (2.375)	14	0.44164
	6	1.74	87.5	28.9	1.897 (2)	14.4	0.35778
	7	1.80	85	26.2	2.003 (2)	35.1	0.92083
	8	1.77	87	27.8	2.165 (2.14)	49.7	1.40957

Note: Sweat rates in parentheses were measured by the naked eye.

Supplementary references

1. Li Y., Wang B. & Feng W. Chiral polyaniline with flaky, spherical and urchin-like morphologies synthesized in the l-phenylalanine saturated solutions. *Synth. Met.* **159**, 1597-1602 (2009).
2. Mažeikienė R., Niaura G. & Malinauskas A. Raman spectroelectrochemical study on the kinetics of electrochemical degradation of polyaniline. *Polym. Degrad. Stab.* **93**, 1742-1746 (2008).
3. Planes G. A., *et al.* Spectroscopic evidence for intermediate species formed during aniline polymerization and polyaniline degradation. *Phys. Chem. Chem. Phys.* **12**, 10584-10593 (2010).
4. Wang M., *et al.* A wearable electrochemical biosensor for the monitoring of metabolites and nutrients. *Nat. Biomed. Eng.* **6**, 1225-1235 (2022).
5. Roy A. C., Nisha V. S., Dhand C., Ali M. A. & Malhotra B. D. Molecularly imprinted polyaniline-polyvinyl sulphonic acid composite based sensor for para-nitrophenol detection. *Anal. Chim. Acta* **777**, 63-71 (2013).
6. Winter M. & Brodd R. J. What Are Batteries, Fuel Cells, and Supercapacitors? *Chem. Rev.* **104**, 4245-4269 (2004).
7. El Sharif H. F., *et al.* Evaluation of electropolymerized molecularly imprinted polymers (E-MIPs) on disposable electrodes for detection of SARS-CoV-2 in saliva. *Anal. Chim. Acta* **1206**, 339777 (2022).
8. Ermiş N., Uzun L. & Denizli A. Preparation of molecularly imprinted electrochemical sensor for l-phenylalanine detection and its application.

J. Electroanal. Chem. **807**, 244-252 (2017).

9. Dinh H. N., Ding J., Xia S. J. & Birss V. I. Multi-technique study of the anodic degradation of polyaniline films. *J. Electroanal. Chem.* **459**, 45-56 (1998).

10. Yang X., Xie Q. & Yao S. A comparative study on polyaniline degradation by an electrochemical quartz crystal impedance system: electrode and solution effects. *Synth. Met.* **143**, 119-128 (2004).

11. Matsushita M., Kuramitz H. & Tanaka S. Electrochemical oxidation for low concentration of aniline in neutral pH medium: application to the removal of aniline based on the electrochemical polymerization on a carbon fiber. *Environ. Sci. Technol.* **39**, 3805-3810 (2005).

12. Orata D. & Buttry D. A. Determination of ion populations and solvent content as functions of redox state and pH in polyaniline. *J. Am. Chem. Soc.* **109**, 3574-3581 (1987).

13. Bagdžiūnas G. Theoretical design of molecularly imprinted polymers based on polyaniline and polypyrrole for detection of tryptophan. *Mol. Syst. Des. Eng.* **5**, 1504-1512 (2020).

14. Tang W.*, et al.* Touch-Based Stressless Cortisol Sensing. *Adv. Mater.* **33**, 2008465 (2021).

15. Sandoval A. P., Orts J. M., Rodes A. & Feliu J. M. A comparative study of the adsorption and oxidation of L-alanine and L-serine on Au(1 0 0), Au(1 1 1) and gold thin film electrodes in acid media. *Electrochimica Acta*

89, 72-83 (2013).

16. Li H.-Q., Chen A., G. Roscoe S. & Lipkowski J. Electrochemical and FTIR studies of l-phenylalanine adsorption at the Au (111) electrode. *J. Electroanal. Chem.* **500**, 299-310 (2001).

17. Aarab N., *et al.* Theoretical study of the adsorption of sodium salicylate and metronidazole on the PANi. *Mater. Today: Proc.* **22**, 100-103 (2020).

18. Zhong B., Jiang K., Wang L. & Shen G. Wearable Sweat Loss Measuring Devices: From the Role of Sweat Loss to Advanced Mechanisms and Designs. *Adv. Sci.* **9**, e2103257 (2022).

19. Yang Y., *et al.* A laser-engraved wearable sensor for sensitive detection of uric acid and tyrosine in sweat. *Nat. Biotechnol.* **38**, 217-224 (2019).

20. Yu Q., *et al.* Semisynthetic sensor proteins enable metabolic assays at the point of care. *Science* **361**, 1122-1126 (2018).

21. Wentland L., Polaski R. & Fu E. Characterization methods in porous materials for the rational design of multi-step processing in the context of a paper microfluidic phenylalanine test. *Anal. Methods* **12**, 768-780 (2020).

22. Sun B., *et al.* Paper-based biosensor based on phenylalnine ammonia lyase hybrid nanoflowers for urinary phenylalanine measurement. *Int. J. Biol. Macromol.* **166**, 601-610 (2021).

23. Hsu L.-W., *et al.* Simultaneous Determination of l-Phenylalanine, Phenylethylamine, and Phenylacetic Acid Using Three-Color Whole-Cell

Biosensors within a Microchannel Device. *ACS Appl. Bio Mater.* **3**, 5120-5125 (2020).

24. Parrilla M., Vanhooydonck A., Watts R. & De Wael K. Wearable wristband-based electrochemical sensor for the detection of phenylalanine in biofluids. *Biosens. Bioelectron.* **197**, 113764 (2022).

25. Mukasa D.*, et al.* A Computationally Assisted Approach for Designing Wearable Biosensors toward Non-invasive Personalized Molecular Analysis. *Adv. Mater.*, e2212161 (2023).
