# Supplementary material for: Interindividual- and blood-correlated sweat phenylalanine multimodal analytical biochips for tracking exercise metabolism
Source: Nat Commun. 2024 Jan 20;15:624. doi: 10.1038/s41467-024-44751-z (PMC10799919; doi:10.1038/s41467-024-44751-z)
Supplement: Supplementary file 3 — Description of Additional Supplementary Files [file 41467_2024_44751_MOESM3_ESM.pdf]

Description of Additional Supplementary Files

Supplementary Video 1 Water filling process in the chamber.

Supplementary Video 2 Refreshing process in the chamber.

Supplementary Video 3 Sweat monitoring during exercise.

Supplementary Video 4 Visualized sweat flowing during exercise.
