# Supplementary material for: Interindividual- and blood-correlated sweat phenylalanine multimodal analytical biochips for tracking exercise metabolism
Source: Nat Commun. 2024 Jan 20;15:624. doi: 10.1038/s41467-024-44751-z (PMC10799919; doi:10.1038/s41467-024-44751-z)
Supplement: Supplementary file 8 — Reporting Summary [file 41467_2024_44751_MOESM8_ESM.pdf]

nature portfolio

Corresponding author(s): Lili, Wang

Last updated by author(s): Dec 27, 2023

Reporting Summary

Nature Portfolio wishes to improve the reproducibility of the work that we publish. This form provides structure for consistency and transparency in reporting. For further information on Nature Portfolio policies, see our Editorial Policies and the Editorial Policy Checklist.

Statistics

For all statistical analyses, confirm that the following items are present in the figure legend, table legend, main text, or Methods section.

n/a	Confirmed	
☐	☒	The exact sample size (*n*) for each experimental group/condition, given as a discrete number and unit of measurement
☐	☒	A statement on whether measurements were taken from distinct samples or whether the same sample was measured repeatedly
☐	☒	The statistical test(s) used AND whether they are one- or two-sided<br>*Only common tests should be described solely by name; describe more complex techniques in the Methods section.*
☒	☐	A description of all covariates tested
☐	☒	A description of any assumptions or corrections, such as tests of normality and adjustment for multiple comparisons
☐	☒	A full description of the statistical parameters including central tendency (e.g. means) or other basic estimates (e.g. regression coefficient) AND variation (e.g. standard deviation) or associated estimates of uncertainty (e.g. confidence intervals)
☐	☒	For null hypothesis testing, the test statistic (e.g. *F*, *t*, *r*) with confidence intervals, effect sizes, degrees of freedom and *P* value noted<br>*Give P values as exact values whenever suitable.*
☒	☐	For Bayesian analysis, information on the choice of priors and Markov chain Monte Carlo settings
☒	☐	For hierarchical and complex designs, identification of the appropriate level for tests and full reporting of outcomes
☐	☒	Estimates of effect sizes (e.g. Cohen's *d*, Pearson's *r*), indicating how they were calculated

Our web collection on statistics for biologists contains articles on many of the points above.

Software and code

Policy information about availability of computer code

Data collection	Data were collected by the following instruments and their accompanying software: CHI 760E (CH Instruments, USA), Multiskan SkyHigh microplate spectrophotometer (Thermo Fisher Scientific, USA), FTIR-650S (Tianjin Gangdong, China), UV-2600i UV/VIS spectrophotometer (Shimadzu, Japan), and ACQUITY UPLC I-Class system and triple quadrupole mass spectrometer XEVO TQ-S (Waters, USA). Data were also collected by the following commercial software: COMSOL Multiphysics 6.0 and Gaussian 16. The codes used for simulation and data measurement are available from the corresponding author with upon reasonable request.
Data analysis	Data were analyzed via Origin 2018, SPSS Statistics 24, and Microsoft Excel 2019.

For manuscripts utilizing custom algorithms or software that are central to the research but not yet described in published literature, software must be made available to editors and reviewers. We strongly encourage code deposition in a community repository (e.g. GitHub). See the Nature Portfolio guidelines for submitting code & software for further information.

Data

Policy information about availability of data

All manuscripts must include a data availability statement. This statement should provide the following information, where applicable:

- Accession codes, unique identifiers, or web links for publicly available datasets
- A description of any restrictions on data availability
- For clinical datasets or third party data, please ensure that the statement adheres to our policy

All data supporting the findings of this study are available within the article and its supplementary files at https://doi.org/10.6084/m9.figshare.24786486. Any additional requests for information can be directed to, and will be fulfilled by, the corresponding authors.

Research involving human participants, their data, or biological material

Policy information about studies with human participants or human data. See also policy information about sex, gender (identity/presentation), and sexual orientation and race, ethnicity and racism.

Reporting on sex and gender	Gender of all male participants was determined based on self-report. Gender was not a factor considered in the experiment design to avoid gender interference in the results.
Reporting on race, ethnicity, or other socially relevant groupings	The study did not involve race, ethnicity, or other socially relevant groupings
Population characteristics	The male healthy participants were between 23 and 27 years old, and were classified into two group according to their different body mass index (BMI) values. including a lean group with a BMI of 18.5 to 24.9 kg m^-2 and an overweight group with a BMI of 25 to 30 kg m^-2.Exclusion criteria included chronic diseases, medication, obesity, genetic metabolic disorders, and other conditions that could interfere with the study.
Recruitment	Recruitment information was shared by posted notices, word of mouth, and on-line platforms. There were no self-selection biases or other biases. All human subjects provided informed consent when they took part in this research.
Ethics oversight	Ethical Committee of Tianjin Medical University General Hospital

Note that full information on the approval of the study protocol must also be provided in the manuscript.

Field-specific reporting

Please select the one below that is the best fit for your research. If you are not sure, read the appropriate sections before making your selection.

☒ Life sciences ☐ Behavioural & social sciences ☐ Ecological, evolutionary & environmental sciences

For a reference copy of the document with all sections, see nature.com/documents/nr-reporting-summary-flat.pdf

Life sciences study design

All studies must disclose on these points even when the disclosure is negative.

Sample size	For the on-body evaluation of the wearable system, 16 participants were recruited (8 lean subjects and 8 overweight subjects), which were sufficient for statistical analysis and comparison between different groups. The sample size was chosen as a small-scale population study. For the sweat-serum phenylalanine correlation study, two representatives from these two groups were recruited and suitable for a pilot study of our system. Sample sizes were chosen on the basis of previous experience and other publications(Nature Communication 2021, 12, 1823), as well as literature standards for proof-of-concept experiments.
Data exclusions	No data were excluded.
Replication	The study validated the sweat multimodal sensing of phenylalanine and chloride concentrations, as well as sweat loss (volume and rate). The result was dependent on the body condition for each experiment session and was thus not replicated. However, for all the on-body evaluation of our wearable systems, the measurements were taken from sixteen participants to demonstrate repeatability. Among them, the sweat test was repeated three times continuously using the wearable system. Moreover, All attempts at replication were successful when following the device-fabrication process described in this paper.
Randomization	The human participants for each experiment were selected randomly. They were classified into two group according to their different body mass index (BMI) values. including a lean group with a BMI of 18.5 to 24.9 kg m^-2 and an overweight group with a BMI of 25 to 30 kg m^-2.
Blinding	No blinding experiments were carried out. All the participants were only told the experiments process, while did not involved in data analysis. Binding to the studies was not required because there was no subjective data and questionnaires related to subjects need to be recorded.

Reporting for specific materials, systems and methods

We require information from authors about some types of materials, experimental systems and methods used in many studies. Here, indicate whether each material, system or method listed is relevant to your study. If you are not sure if a list item applies to your research, read the appropriate section before selecting a response.

Materials & experimental systems

n/a	Involved in the study
☐	☒ Antibodies
☒	☐ Eukaryotic cell lines
☒	☐ Palaeontology and archaeology
☒	☐ Animals and other organisms
☒	☐ Clinical data
☒	☐ Dual use research of concern
☒	☐ Plants

Methods

n/a	Involved in the study
☒	☐ ChIP-seq
☒	☐ Flow cytometry
☒	☐ MRI-based neuroimaging

Antibodies

Antibodies used	Rabbit anti-L-Phenylalanine antibody was used in the commercial L-Phenylalanine ELISA kit (Immusmol, France) for standardized phenylalanine quantification. Antibody validation was performed by the individual manufacturer and their data are available on the manufacturers' website.
Validation	The L-Phenylalanine ELISA kit was used based on the manufacturer's instruction (https://www.immusmol.com/wp-content/uploads/2022/02/IFU_IS-I-1700R_V3.0.pdf) and Safety Data Sheet (https://www.immusmol.com/wp-content/uploads/2022/02/SDS-IS-I-04_05_11_12_13_17.pdf).

Plants

Seed stocks	n/a
Novel plant genotypes	n/a
Authentication	n/a
